# Menstrual characteristics and dysmenorrhea among Palestinian adolescent refugee camp dwellers in the West Bank and Jordan: a cross-sectional study

**DOI:** 10.1186/s13690-023-01059-6

**Published:** 2023-03-30

**Authors:** Rula Ghandour, Weeam Hammoudeh, Hein Stigum, Rita Giacaman, Heidi Fjeld, Gerd Holmboe-Ottesen

**Affiliations:** 1 Institute of Community and Public Health, Birzeit University, Said Khoury Building for Development Studies, Birzeit, P.O.Box 14, occupied Palestinian territory; 2 Department of Community Medicine and Global Health, University of Oslo, Oslo, Norway

**Keywords:** Menstruation, Menstrual characteristics, Dysmenorrhea, Adolescent refugees, Jordan, West Bank of the occupied Palestinian territory

## Abstract

**Background:**

Women and girls experience menstruation throughout their reproductive years. Normal adolescent menstrual cycles gauge current and future reproductive health. Dysmenorrhea (painful menstruation) is the most prevalent menstrual disturbance in adolescents that can be debilitating. This study examines the menstrual characteristics of adolescent girls living in Palestinian refugee camps in the West Bank of the Israeli-occupied Palestinian territory and Jordan, including estimates of dysmenorrhea levels and associated factors.

**Methods:**

A household survey of 15 to 18-year-old adolescent girls was conducted. Trained field workers collected data on general menstrual characteristics and dysmenorrhea level using Working ability, Location, Intensity, Days of pain Dysmenorrhea scale (WaLIDD), in addition to demographic, socio-economic, and health characteristics. The link between dysmenorrhea and other participant characteristics was assessed using a multiple linear regression model. Additionally, data on how adolescent girls cope with their menstrual pain was collected.

**Results:**

2737 girls participated in the study. Mean age was 16.8 ± 1.1 years. Mean age-at-menarche was 13.1 ± 1.2; mean bleeding duration was 5.3 ± 1.5 days, and mean cycle length was 28.1 ± 6.2 days. Around 6% of participating girls reported heavy menstrual bleeding. High dysmenorrhea levels were reported (96%), with 41% reporting severe symptoms. Higher dysmenorrhea levels were associated with older age, earlier age-at-menarche, longer bleeding durations, heavier menstrual flow, skipping breakfast regularly, and limited physical activity patterns. Eighty nine percent used non-pharmacological approaches to ease menstrual pain and 25% used medications.

**Conclusion:**

The study indicates regular menstrual patterns in terms of length, duration, and intensity of bleeding and a slightly higher age-at-menarche than the global average. However, an alarmingly high prevalence of dysmenorrhea among participants was found that tends to vary with different population characteristics, some of which are modifiable and can be targeted for better menstrual health.This research emphasizes the need for integrated efforts to assist adolescents with menstrual challenges such as dysmenorrhea and irregular periods to achieve informed recommendations and effective actions.

**Supplementary Information:**

The online version contains supplementary material available at 10.1186/s13690-023-01059-6.

## Background

Menstruation is a natural biological process that all girls and women undergo during their reproductive lives [1]. Normal menstrual cycles are a sign of good health and an integral part of reproductive health and general well-being [2]. Age at menarche, or the age at the first menstrual cycle, is a main milestone for adolescent girls marking the onset of puberty and is also an important indicator of health [1, 3]. Identifying regular and irregular menstruation is crucial, especially that some irregularities are linked to underlying causes, such as endometriosis and other ovarian or adrenal disorders. Awareness of these irregularities can help in early diagnosis and treatment [4]. The American College of Obstetricians and Gynecologists and the American Academy of Pediatrics have endorsed monitoring menstrual regularity patterns as a vital sign of health for adolescent girls. They also recommend that preparation and education should target health care providers and caregivers with accurate information about regularity patterns and instructions on what to do in irregular situations [4].

Dysmenorrhea, or painful menstruation, is the most prevalent menstrual irregularity among adolescent girls [5, 6]. The prevalence of dysmenorrhea can reach up to 90%, with wide variations between different studies and within different populations [5]. Dysmenorrhea is classified as either primary or secondary. Around 90% of adolescents have primary dysmenorrhea without underlying pelvic pathology [7], and pain is usually caused by excessive prostaglandin production (pain neurotransmitter in the uterus) [8]. Secondary dysmenorrhea is frequently associated with underlying pelvic diseases that require medical care, such as endometriosis, ovarian cysts, or fibroids [5].

Dysmenorrhea is a debilitating event that usually affects women’s and girls’ well-being, productivity, and quality of life [6, 8]. In the literature, primary dysmenorrhea is consistently the leading cause of school absenteeism for adolescent girls and negatively affects their participation in curricular and non-curricular activities [5, 8, 9]. Furthermore, dysmenorrhea can be associated with adverse health conditions in adult life, including endometriosis, cardiovascular disease, diabetes, and breast cancer [10].

To cope with dysmenorrhea, women and girls usually self-medicate or use non-pharmacological approaches - especially in resource-poor environments - and rarely seek medical attention, perceiving this suffering as expected [11]. Non-pharmacological approaches are reported to be more widely used by adolescent girls believing in their safety. On the other hand, self-medication is frequently insufficient, inadequate, or inefficient, particularly in cases of secondary dysmenorrhea where additional medical care is required [7, 12].

In recent years, menstrual health research has gained greater momentum in an effort to understand its various dimensions and how they might be addressed at various levels and in diverse circumstances. Nonetheless, efforts have been scattered, and definitions are inconsistent. For example, literature and research from low-income and low-middle-income countries in South Africa and East Asia focus on water, sanitation, and hygiene (WASH). In contrast, high-income countries emphasize menstrual equity and period poverty, which demand equitable access to menstrual education, hygienic sanitary products, and infrastructure [13, 14]. In response to these fragmented initiatives and consolidating them, the Global Menstrual Collective was founded in 2019 to integrate work on menstrual health and provide a unified definition for policy, practice, and research [15]. Furthermore, there is a global call to integrate menstrual health into broader sexual and reproductive health and rights, aiming to enable all women and girls to experience healthy menstruation with dignity [2].

To our knowledge, current research on menstrual health from the Middle East and North Africa (MENA) area, including Arab countries, has been limited and insufficient. In this paper, we aim to describe adolescent girls’ menstrual characteristics in a specific context: Palestinian refugee camps in Jordan and the Israeli-occupied West Bank. We also aim to assess the prevalence of dysmenorrhea and its different dimensions and to identify the main associated factors, including demographic, socio-economic, behavioral, and menstrual characteristics. Finally, we aim to provide insight into adolescent girls’ menstrual management practices.

## Methods

The results presented here are part of a larger mixed methods study conducted in Palestinian refugee camps in the West Bank of the Israeli-occupied Palestinian territory (oPt) and Jordan. The study addressed adolescent girls’ general health status and needs, including nutrition, reproductive health, and mental health [16, 17]. This paper focuses on menstrual patterns and characteristics. It also measures the prevalence of dysmenorrhea, its different dimensions, and its management strategies. The study is based on cross-sectional data collected through a household survey conducted between June and September 2019.

### Study setting

This study targeted 15–18 years old girls living in any of the Palestinian refugee camps in the West Bank of the oPt or Jordan. Established in 1949, the Palestine refugee camps are long-lasting camps where the adolescent populations are third or fourth-generation refugees. Palestinian refugees in the West Bank are internally displaced refugees living within territories under Israeli military occupation, while Palestinian refugees in Jordan have been dispossessed, dispersed, and externally displaced from their homeland due to the 1948 and 1967 Arab - Israeli wars [18]. Palestinian refugee camps are densely populated, overcrowded, impoverished, with inadequate infrastructure, and in general, dominated by patriarchal relationships and gender norms, and characterized by conservative social dynamics [18–20]. The long-lasting existence of refugee camps has shifted the refugees’ needs from immediate requirements for food and shelter to improved living conditions [21]. There are nineteen Palestine refugee camps in the West Bank and ten in Jordan [22].

### Sample and sampling procedure

To ensure representativeness, we collected data from all 29 Palestinian refugee camps in the West Bank and Jordan. The sample was a stratified random sample with a proportional selection of participants according to refugee camp population size. This was calculated for the West Bank and Jordan independently based on a standard stratified random sample equation and then corrected for design effect. The minimum required sample size calculated was 2428 adolescent girls. The final survey sample included 2949 girls. The inclusion criteria for the analysis presented in this study were girls aged between 15 and 18 years, residing in any refugee camp in the West Bank or Jordan at the time of the survey, never married, and have experienced at least one menstrual episode. Any girl who did not meet the inclusion criteria was excluded. After applying the inclusion and exclusion criteria, the final empirical sample consists of 2737 girls (see supplement 1 for detailed calculations and sample flowchart).

Data were collected using a random walk door-to-door technique [23] by trained field workers from the local community (women camp dwellers or familiar with camp life). We targeted all households at the refugee camps and recruited participants from households with adolescent girls. Consent was obtained first from the mother or caregiver and then from the adolescent girl(s) in the household. If more than one girl within the same household met the inclusion criteria, they were all invited to participate. Each interview was done separately and in private to ensure confidentiality. If the mother or caregiver was unavailable for interview, the field workers made an appointment and tried up to 3 times to reach her before dropping the girl from the sample.

### Tools and data collection

The survey instrument included a roster form with demographic and socio-economic information, which the girls’ mothers completed. A different form covering demographic, socio-economic, health, health-related practices, and menstrual health information was used for the girls. The data was collected using electronic tablets and transferred to a local secure server.

### Study variables

Demographic and socio-economic information included: the girls’ age, country, camp locality, and whether the household consisted of a nuclear family (i.e., only parents and children) or an extended family (additional relatives residing in the dwelling). The crowding ratio at the household level was measured by dividing the number of people living in the household by the number of rooms in the residence. The standard of living index was calculated as a proxy for economic status. To calculate the index, each household was questioned on the availability of a set of amenities (24 items). These items were based on what is usually asked in health and population surveys by the Palestinian Central Bureau of Statistics (PCBS) in the West Bank [24] and the Department of Statistics (DoS) in Jordan [25]. Then, a score was calculated using factor analysis in which the components were forced into a single factor, and scores were obtained using the regression approach. The scale was then modified to give a score range of 0-100 and recoded into 3 categories.

Menstrual regularity patterns were collected by asking the girls directly about their age-at-menarche (AAM), cycle frequency (average length between 2 cycles), bleeding duration (number of bleeding days), and heaviness of bleeding. The heaviness of bleeding was measured using a proxy question i.e. if blood leaked through the girls’ clothes during the first two days of the period using a Likert scale for the response [26–28]. Age at menarche (AAM) was considered normal if it occurred between the age of 8 years to less than 17 years old and was then categorized into *normal early* (8 to < 12 years old), *normal* (12-14 years old) and *normal late* (between 14 and 17 years old) [34]. Time since menstruation was calculated by subtracting AAM from the present age. The bleeding duration was considered normal if it was between 3 and 7 days, short periods < 3 days and long periods if bleeding continues to more than 7 days [26, 28]. Bleeding frequency was considered normal if it ranged between 21 and 45 days, frequent if it was less than 21 days and infrequent if more than 45 days [26].

The main outcome of this study was dysmenorrhea. The epidemiological literature measures dysmenorrhea using either a uni-dimensional question/score or a multi-dimensional scale. A major criticism of uni-dimensional measures is that adolescents cannot appropriately report menstrual discomfort during the early phases of menstruation since they are still learning about their bodies and their changes, thus necessitating a multi-dimensional scale [6, 7, 29, 31, 30]. Thus, in our study, we measured dysmenorrhea using the Working ability, Location, Intensity, Days of pain Dysmenorrhea (WaLIDD) scale developed by Teherán et al. [32]. It relied on measuring four dimensions of dysmenorrhea, with each given a score of 0–3; and a combined score from 0 to 12. The total score was then categorized into four different degrees of dysmenorrhea (0 = none, 1–3 = mild, 4–7 = moderate, and 8–12 = severe). The categories for the WaLIDD scale were used for description, and the continuous score was used in the bivariate and multivariate analyses.

### Statistical analysis

We used the continuous dysmenorrhea score as the dependent variable in the bivariate and multivariate analyses. We assessed statistical significance in the bivariate analysis using t-test, one-way ANOVA, and Pearson correlation coefficient. A multiple linear regression model was conducted to test for significantly associated factors with dysmenorrhea. We examined the assumptions of the standard regression model. The constant error variance assumption was examined by the heteroscedasticity test, that was significant (p-value < 0.001) and suggested heteroscedasticity in our model. However, this did not appear to be problematic. We ran a robust model analysis and we found that robust-based standard errors were similar to normal based standard errors. To identify influential outliers, we plotted a chart of leverage vs. residuals. We eliminated the three most influential points and compared the resulting model to the original; we found no significant differences, indicating no influential outliers. Additionally, we looked for potential interactions or collinearities, but none were detected.

### Ethical considerations

The study was approved by the Research Ethics Committee at Birzeit University (reference number 171,114) in December 2017 and by the Regional Committee for Medical and Health Research Ethics (REC) in Norway (reference number 2018/2206) in June 2019. Written consent for participation was obtained from the girls and their female caregivers in the same household. Data collection took place in a private space, ensuring girls’ privacy and confidentiality. Data were anonymized before starting the analysis.

## Results

The survey included 2737 girls, with a response rate of 81% at the household level. For adolescent girls within the accepting households, response rate was 97%. The average age for participating girls was 16.8 ± 1.1 years. The mean number of members per household was 7 ± 2, with around 60.4% of the participants reporting being the only adolescent girl (15-18 years old) in the household, while the rest had adolescent sisters within this age range. Half of the girls were living in refugee camps in Jordan and the other half in the West Bank, with nearly three-quarters residing in camps close to urban areas, 22.8% in camps close to rural areas, and only 3.6% close to Bedouin communities. Most participating girls lived in nuclear families (95.8%), and 19.4% lived in crowded households. The mean standard of living score (out of 100) was 37.5 ± 16.8, and the median was 33.2. Still, 36.4% of the girls reported having good to very good economic status compared to those around them, and 51.1% reported having fairly good economic status (Table 1).

**Table 1:** Sample characteristics of adolescent girls 15-18 years old living in Palestinian refugee camps in the West Bank and Jordan (N = 2737)

Demographic and socio-economic characteristics		N	%
Girls’ age (years) N = 2737	15	867	31.7
	16	691	25.2
	17	686	25.1
	18	493	18.0
Family type N = 2654	Nuclear family	2543	95.8
	Extended family	111	4.2
Crowding ratio N = 2652	Not crowded	2137	80.6
	Crowded	515	19.4
Standard of living index score N = 2737	Low (less than 30)	1040	38.0
	Moderate (31–60)	1379	50.4
	High (> 60)	318	11.6
Reported economic status N = 2737	Bad to very bad	343	12.5
	Fair	1,399	51.1
	Good to very good	995	36.4
Number of adolescents girls 15-18 years old at the household N = 2737	1	1,654	60.4
	More than 1	1,083	39.6
Nature of camp community N = 2737	Urban	2,013	73.5
	Rural	625	22.8
	Bedouin	99	3.6
Country N = 2737	West Bank	1,364	49.8
	Jordan	1,373	50.2
Health and healthy behaviors		N	%
Skipping breakfast N = 2721	Rarely	812	29.8
	Sometimes	869	32.0
	Always	1,040	38.2
On diet for weight loss N = 2699	No	2379	88.1
	Yes	320	11.9
Physically active for at least 1 h/day during a regular week N = 2736	0 days	193	7.0
	1–5 days	1,641	60.0
	6–7 days	902	33.0
Self-rated health N = 2736	Very bad to bad	118	4.3
	Neither good nor bad	281	10.3
	Good to very good	2,337	85.4

At the time of the survey, more than one-third of the girls reported regularly skipping breakfast, and 11.9% were on a weight-loss plan. Furthermore, 33% reported engaging in at least 1 h of physical activity daily, 60% were active for at least 1 h once per 1 to 5 days, and 7% were never physically active. Despite this, 85.4% of girls reported their health as good to very good (Table 1).

### Menstrual regularity patterns

The average AAM was 13.1 ± 1.2 years, while average years passed since menarche was 3.6 ± 1.5. The proportion of girls who reached menarche by the age of 14 years was 87.7%. The majority reported regular bleeding duration ranging between 3 and 7 days (94.2%), with 1.5% reporting shorter periods and 4.3% reporting longer periods. Overall, the average number of bleeding days was 5.3 ± 1.5, and cycle length was 28.1 ± 6.2 days, whereas 3.6% were unable to identify their cycle length either because it was irregular or because they did not know how to assess cycle length. Finally, 6.3% percent mentioned having heavy bleeding. (Table 2).

**Table 2:** Menstrual characteristics for adolescent girls 15–18 years living in Palestinian refugee camps in the West Bank and Jordan (N = 2737)

		N	%
Age at menarche Mean (13.1 ± 1.2) N = 2737	Normal early (< 12 years old)	204	7.5
	Normal (12–14 years old)	2195	80.2
	Normal late (> 14 and < 17 years old)	338	12.3
Time since menarche Mean (3.6 ± 1.5) N = 2737	0–2 years	406	14.8
	> 2–4 years	1,232	45.0
	> 4 years	1,099	40.2
Bleeding duration Mean (5.3 ± 1.5) N = 2729	Regular (3 to 7 days)	2570	94.2
	Short period (< 3 days)	41	1.5
	Long period (> 7 days)	118	4.3
Bleeding frequency Mean (28.1 ± 6.2)* N = 2729	Normal short 21–28 days)	1,586	58.1
	Normal long (29–45 days)	912	33.4
	Frequent (< 21 days) days	71	2.6
	Infrequent (> 45 days)	62	2.3
	Irregular/ do not know	98	3.6
Heaviness of bleeding (Blood leaks through clothes) N = 2732	Never	1668	61.1
	Rarely	440	16.1
	Sometimes	451	16.5
	Mostly/ always	173	6.3

* *Mean calculation excluded those with irregular periods or those who do not know how to calculate*

### Dysmenorrhea prevalence and associated factors

**Fig. 1:**
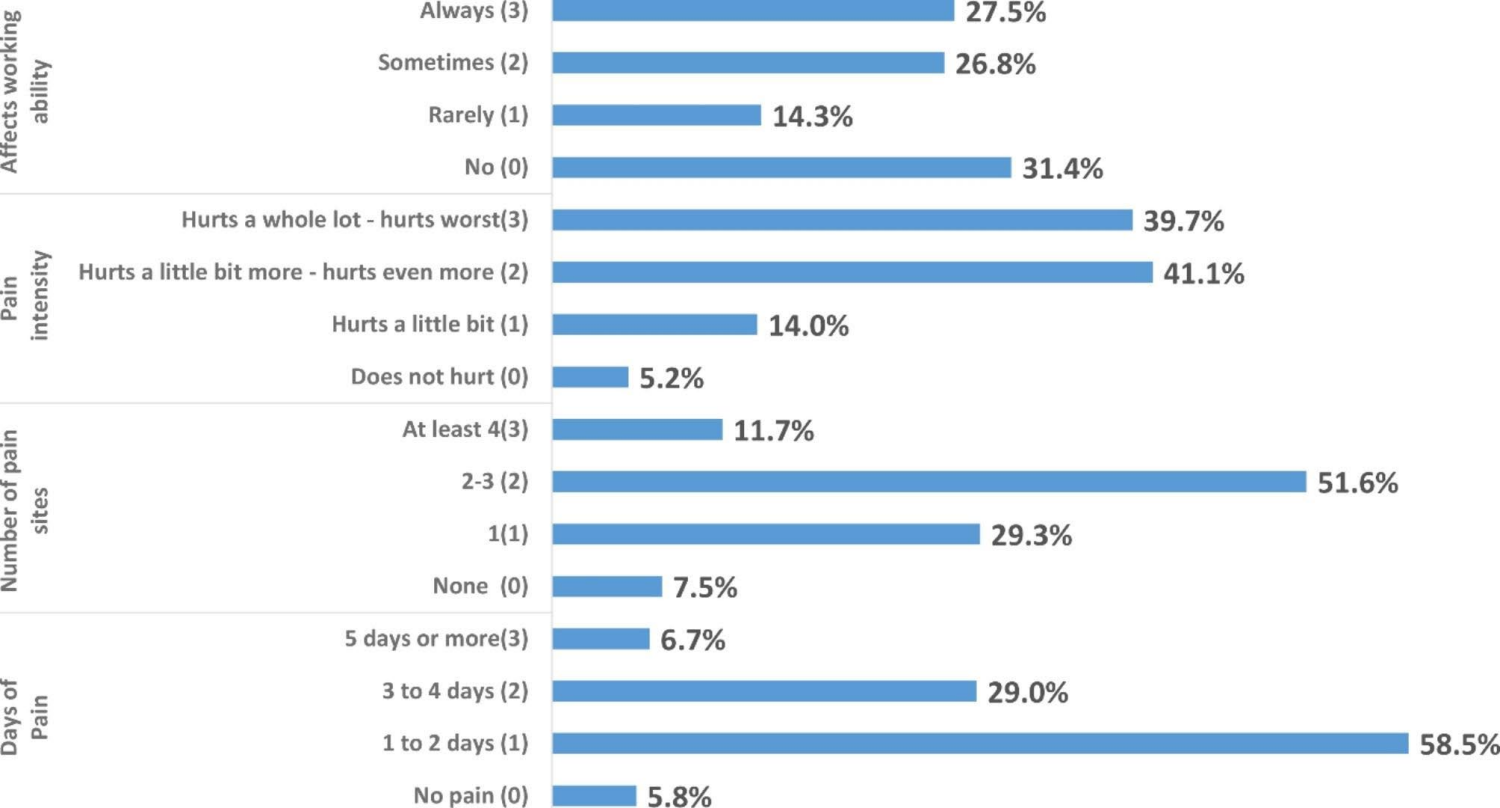
Working ability, Location, Intensity, and Days of Pain Dysmenorrhea score (WaLIDD) four main dimensions* *Each dimension has four levels rated 0 through 3. All scores contribute to a total WaLIDD score between 0 and 12

**Fig. 2:**
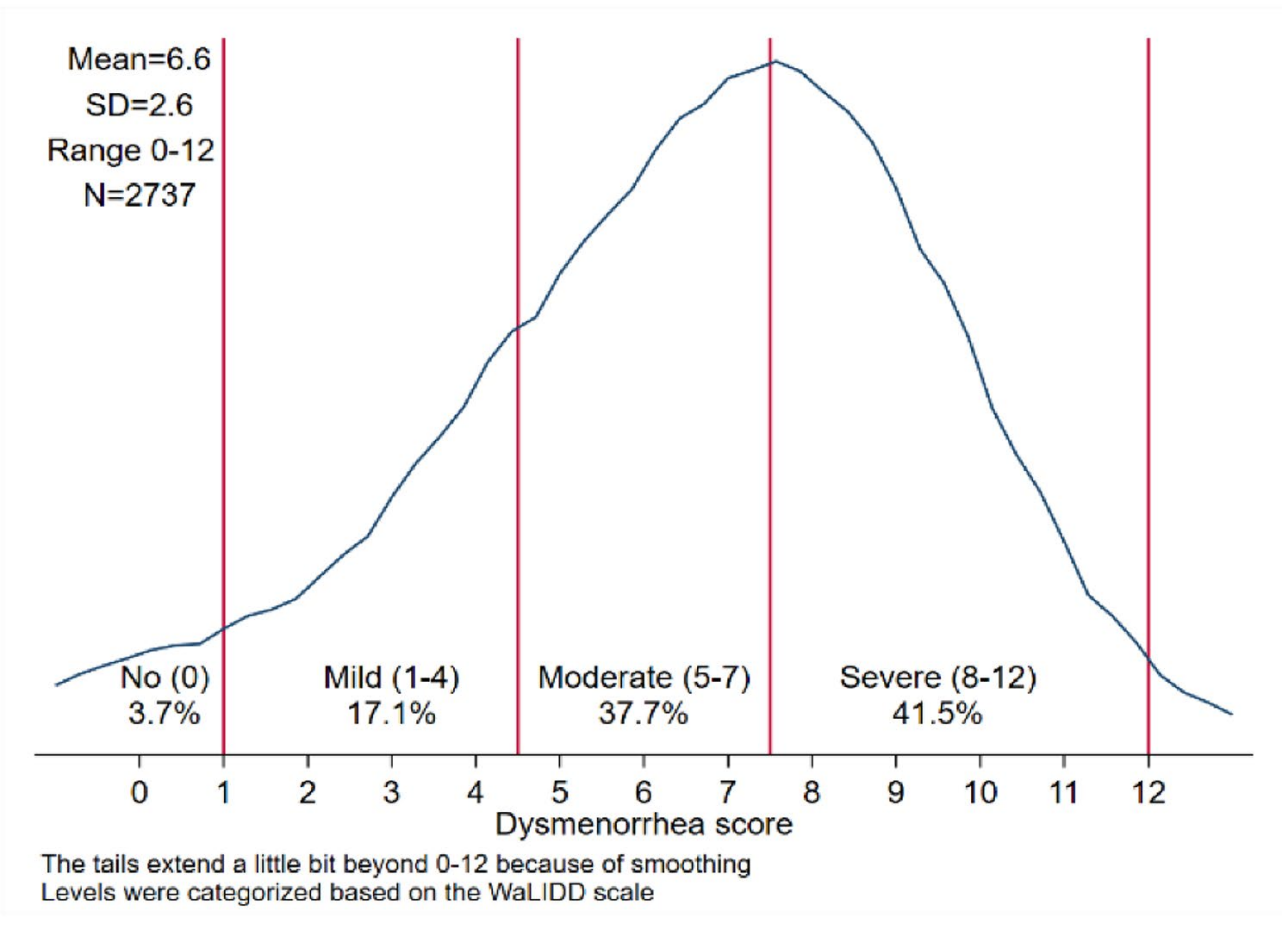
WaLIDD score distribution among adolescent girls 15–18 years living in Palestinian refugee camps in the West Bank and Jordan

Concerning dysmenorrhea, the internal consistency of the WaLIDD multi-dimensional scale items was high, with Cronbach’s alpha = 0.75. The WaLIDD scale indicated that the prevalence of dysmenorrhea was extremely high (96.3%). According to WaLIDD scores, 17.1%, 37.7%, and 41.5% of participants, respectively, exhibited mild, moderate, and severe dysmenorrhea. The mean score for dysmenorrhea (MDS) was 6.6 ± 2.6. Figure 1 shows the distribution of the four dimensions of the WaLIDD scale, whereas Fig. 2 shows the distribution of the WaLIDD score (0–12) divided into four levels of pain intensity (none, mild, moderate, and severe) according to Teherán and colleagues’ scoring classification [32].

Given these high rates of dysmenorrhea according to the WaLIDD score, we examined the distribution of dysmenorrhea among different subgroups and how scores varied by different population characteristics. Based on the bivariate analysis and the adjusted multiple linear regression model, the mean dysmenorrhea score (MDS) varied significantly across selected demographic and socio-economic characteristics. MDS increased with older age (0.28 points higher MDS with each 1-year increase in age, p-value < 0.001) and decreased with having at least one sister within the same age group (0.23 lower MDS, p-value 0.017). Living in refugee camps close to rural areas was linked to lower MDS compared to camps close to urban areas (0.5 points lower MDS, p-value = 0.001). Girls living in refugee camps in Jordan reported higher MDS than the West Bank (0.35 higher MDS, p-value < 0.001). Reported household economic status was significantly associated with MDS in both the bivariate and multivariate analysis; girls reporting good to very good economic status had 0.56 points lower MDS compared to girls who reported their economic status as bad to very bad (p-value 0.001).

Girls who reported regularly skipping breakfast reported 0.48 points higher MDS compared to girls who rarely skipped breakfast (p-value < 0.001), while girls who indicated performing physical activity daily reported 0.52 points lower MDS, which was moderately significant (p-value 0.010). Notably, girls who rated their health as good to very good reported 1.46 lower MDS than those who reported their health as bad to very bad (p-value < 0.001).

The analysis revealed a highly significant association between dysmenorrhea and menstrual characteristics, including AAM, cycle duration, and heaviness of bleeding, but not with menstrual cycle frequency. MDS decreased with increasing AAM. With each one-year increase in AAM, there was 0.15 MDS reduction (p-value < 0.001). Furthermore, with each one-day increase in bleeding days, the MDS increased by 0.18 (p-value < 0.001). Finally, MDS increased significantly with the increased heaviness of bleeding. For girls who reported blood leaking through their clothes during menstruation, MDS increased by 0.55, 0.76, and 1.21 points, respectively for the rarely, sometimes, and always categories compared to girls who said this had never happened (p-value < 0.001). (Table 3)

**Table 3:** Linear regression for association between WaLIDD score and associated factors in adolescent girls 15–18 years old living in Palestinian refugee camps in the West Bank and Jordan (N = 2737)

			Bivariate analysis				Linear regression		
			Mean dysmenorrhea score	Unadjusted mean difference	Unadjusted p-value		Ajusted mean difference	Adjusted P-value	Adjusted 95% CI
Age (years)^1^				0.26^2^	< 0.001		0.28^2^	< 0.001	( 0.20,0.36 )
Number of adolescent girls at the same household	One girl		6.74	*ref*			*ref*		
	More than one girl		6.40	-0.34	0.001		-0.23	0.017	( -0.49,-0.04 )
Reported economic status compared to others	Bad to very bad		7.22	*ref*			*ref*		
	Fair		6.77	-0.45	0.004		-0.25	0.110	( -0.56,0.04 )
	Good to very good		6.16	-1.06	< 0.001		-0.56	0.001	( -0.9,-0.27 )
Standards of living index score^1^				-0.01^2^	< 0.001		0.0	0.646	--
Country	West Bank		6.44	*ref*			*Ref*		
	Jordan		6.77	0.33	0.001		0.35	0.001	( 0.15,0.56 )
Nature of camp community	Urban		6.80	*ref*			*ref*		
	Rural		5.92	-0.89	0.000		-0.50	< 0.001	( -0.73,-0.27 )
	Bedouin		6.84	0.04	0.896		-0.34	0.196	( -0.87,0.18)
Skipping breakfast	Rarely		6.21	*ref*			*ref*		
	Sometimes		6.62	0.41	0.001		0.29	0.015	(0.06,0.53)
	Always		6.88	0.66	< 0.001		0.48	< 0.001	(0.25,0.71)
On a diet to lose weight	No		6.56	*ref*			*ref*		
	Yes		6.93	0.37	0.017		0.10	0.498	( -0.19,0.39 )
Generally, the number of days of being physically active per week	0 days per week		7.17	*ref*			*ref*		
	1–5 days per week		6.76	-0.41	0.039		-0.19	0.336	( -0.57,0.19 )
	6–7 days per week		6.19	-0.98	< 0.001		-0.52	0.010	( -0.92,-0.13 )
Self-rated health	Bad to very bad		8.27	*ref*			*ref*		
	Neither good nor bad		7.62	-0.65	0.022		-0.55	0.048	( -1.09,-0.01 )
	Good to very good		6.39	-1.88	< 0.001		-1.46	< 0.001	( -1.94,-0.99 )
Age at menarche (years)^1^				-0.19^2^	< 0.001		-0.15^2^	< 0.001	( -0.23,-0.07 )
Bleeding duration				0.25^2^	< 0.001		0.18^2^	< 0.001	( 0.12,0.25)
Bleeding frequency	Normal short 21 to 28 dyas		6.56	*ref*			*ref*		
	Normal long 29 to 45 days		6.61	0.05	0.668		0.11	0.298	(-0.10,0.31)
	Frequent (less than 21 days)		7.38	0.82	0.011		0.56	0.060	(0.02,1.15)
	Infrequent (more than 45 days)		6.79	0.23	0.508		0.05	0.877	(-0.58,0.68)
	Irregular or do not know		6.32	-0.25	0.364		-0.32	0.228	(-0.84,0.20)
Heaviness of bleeding (blood usually leaks through clothes)	Never		6.20	*ref*			*ref*		
	Yes, rarely		6.92	0.72	< 0.001		0.55	< 0.001	( 0.29,0.82 )
	Yes, sometimes		7.25	1.05	< 0.001		0.76	< 0.001	( 0.50,1.03 )
	Yes, most of the time or always		7.93	1.73	< 0.001		1.21	< 0.001	( 0.81,1.61 )

### Dysmenorrhea management

**Fig. 3:**
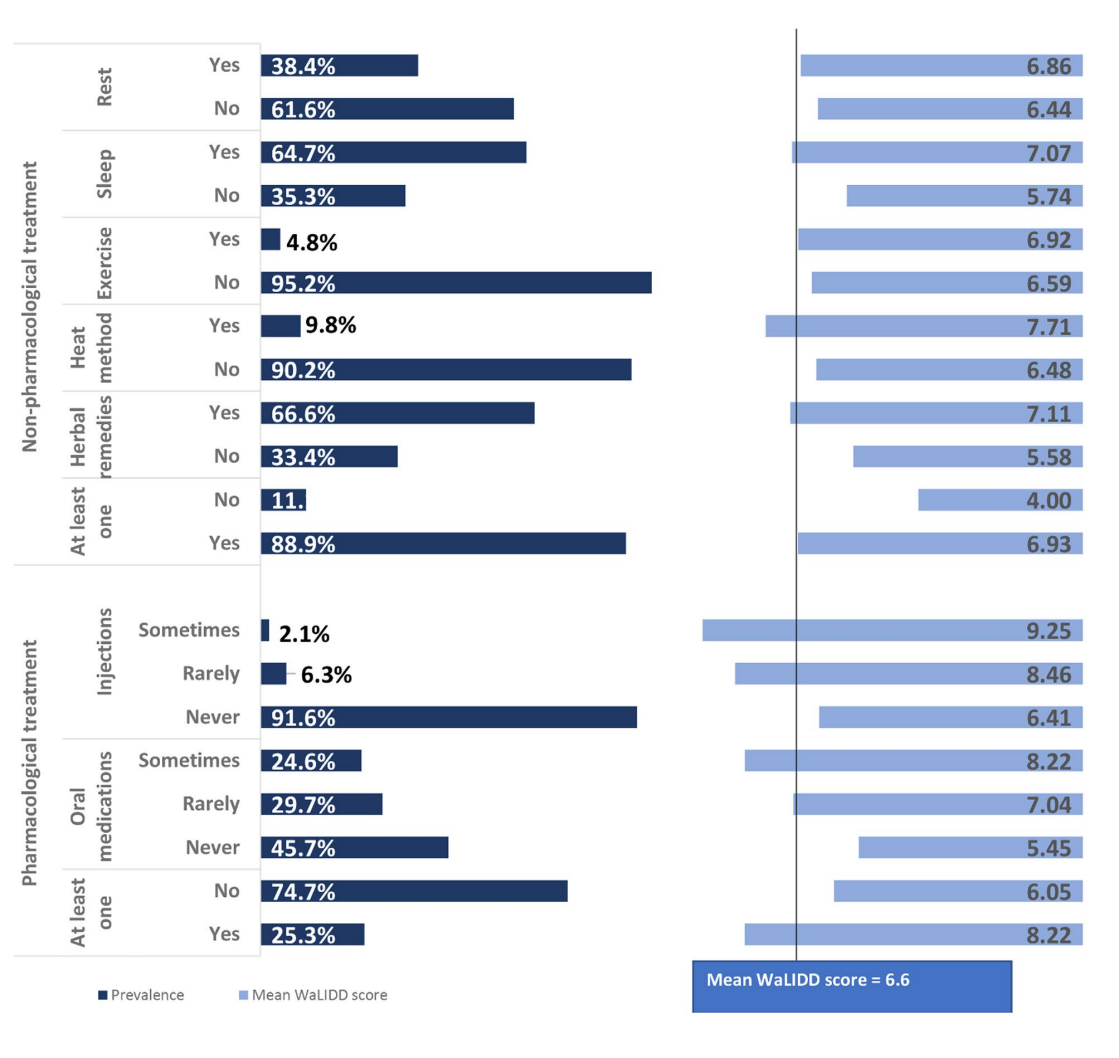
Reported dysmenorrhea management practices by WaLIDD scores for adolescent girls 15–18 years old living in Palestinian refugee camps in the West Bank and Jordan (N = 2737)

We checked the girls’ dysmenorrhea management strategies and if they differed by pain intensity. We found that 88.9% reported utilizing at least one nonpharmacologic management strategy including hot water bag, exercise, sleep, or herbal tea, whereas only 25.3% percent said they occasionally used pharmaceuticals. Figure 3 reflects the distribution and types of pharmacological and non-pharmacological management strategies adolescent girls used to ease their dysmenorrhea. Girls who reported using any management strategy consistently reported higher MDS than those who did not. (Fig. 2)

## Discussion

This study sheds light on the main menstrual health characteristics of adolescent girls living in long-lasting Palestine refugee camps in the West Bank and Jordan. It reveals a high percentage of regular periods in terms of length, duration, and intensity of bleeding and a slightly higher AAM than the global average, which ranges between 12 and 13 years-old [28]. Most importantly, our study revealed an alarmingly high prevalence of dysmenorrhea among the participating girls, which varied by different population characteristics and management strategies. Higher levels of dysmenorrhea were associated with older age, earlier age at menarche, longer bleeding durations, heavier menstrual flow, regularly skipping breakfast, and low levels of physical activity. In addition, the mean dysmenorrhea score was higher for those who utilized dysmenorrhea management measures, whether pharmacological or non-pharmacological.

Menstrual regularity during adolescence is an important indicator of good health. Evidence shows that irregular periods during adolescence are linked to a higher risk of chronic disease and premature mortality [35]. However, menstrual irregularities in our study were not as prevalent as reported in other comparable studies. Our data indicated that only 6–8% of the participating girls had irregular periods in terms of duration, frequency, or intensity of bleeding where other regional studies have found higher rates. For example, studies from Iraq, Jordan and Lebanon showed that around one-third of their respondents had irregular periods [36–38]. The same applies to the heaviness of bleeding, which was found to be much higher in Lebanon (27%) [37] and Kuwait (31%) [30]. Given the variation in tools and definitions employed in different studies, we do not know how comparable these numbers are.

Beyond regularity patterns, dysmenorrhea is the most prevalent menstrual abnormality or disorder among adolescents [5]. A recent meta-analysis of dysmenorrhea among students determined that the prevalence ranged from 24 to 94%, with an average of 66% and 8% reporting severe symptoms [8]. In our study, dysmenorrhea was reported by 96% of the participating girls, and 41% reported it as severe. Other studies have also reported similarly high levels among school girls from the region. For example, in a study in Morocco, 78% of participants reported dysmenorrhea, of whom 45% had severe symptoms [39]. In Iraq, the rate of dysmenorrhea was found to be 89%, and around 40% percent reported severe symptoms [36]; in Kuwait, it was 86% [30]; in Oman, 94%; in Saudi Arabia 74%, and in Egypt 75% [39]. Rates among school girls are very high in high-income countries, with at least three-quarters of adolescent girls reporting dysmenorrhea and 15-20% reporting it as severe [7, 39, 40]. Only a few such studies have been carried out in Palestine and Jordan. However, two studies of university students revealed that 90% of Jordanian [12] and 85% of Palestinian students [41] routinely experienced dysmenorrhea.

Although evidence is available on dysmenorrhea levels from several countries, these numbers should be treated with caution and might not be comparable. It has consistently been reported that measuring dysmenorrhea is problematic because no universal measurement tool has been agreed upon, leaving a wide range of variation in prevalence [6, 8]. Furthermore, it was reported that dysmenorrhea is not only about pain but may also be accompanied by other symptoms that can have debilitating effects on girls’ daily life and sometimes a need for medical attention [31, 31]. Thus, we believe that assessing dysmenorrhea should rely on self-report multi-dimensional measurements such as the one we used in this study [32]. It can be argued that pain self-reports are subjective and, therefore, inaccurate [5, 6]. In contrast, the subjective measure can be understood as a behavioral manifestation of pain perception. Pain is a bio-psycho-social experience and is affected by non-physiological factors, including personal, cognitive, emotional and psychological factors that might affect pain intensity and vary within different population groups and individuals [31, 42–44]

In our study, dysmenorrhea tends to vary with different population characteristics that are worth understanding. We found that dysmenorrhea prevalence rose with age, contrary to previous reports of an inverse relationship [45]. However, this may not be unusual given the target age group in our study. Dysmenorrhea is less common during the first two years following menarche when menstruation is anovulatory [6, 7]. A recent meta-analysis of students found that those older than 20 years had a higher risk of dysmenorrhea than those younger [8]. A survery of 12-20-year-old Moroccan students confirmed this finding [39].

Location of residence seems to affect the level of dysmenorrhea, although the mechanisms behind this are unknown. It has previously been linked to socio-economic level and proximity to health and educational resources [6, 46]. In our study, girls from the Jordan reported slightly higher levels of dysmenorrhea than those from the West Bank. Additionally, those residing in rural rather than urban refugee camps reported lower scores; this distinction between rural and urban areas has been reported previously in Jordan [12]. Worth noting that although previous research has focused on menstrual health in refugee and emergency settings [47], most literature on dysmenorrhea does not differentiate between refugee camp settings and nonrefugee-camp settings. Hence it is difficult to identify the particularity of the refugee-camp setting for experiences with menstrual pain. Dysmenorrhea is a universal condition affecting girls and women in all societies. However, according to our results, higher levels of severe dysmenorrhea was related to poorer socio-economic conditions and probably due to the chronic stress of living in refugee camps.

Interestingly, having sisters in the same age group (15-18 years old) was linked to lower dysmenorrhea levels in our study, contrary to a study conducted in Kuwait [30]. Few studies have addressed the impact of having sisters with dysmenorrhea beyond genetics and family history. However, in the present case we assume that having sisters can offer social support by sharing information, knowledge, and comfort. This could possibly help ease pain and provide assistance with pain management. These results are consistent with previous research findings, which indicate that social support is linked to lower levels of dysmenorrhea [5, 8, 48, 49].

The association between dysmenorrhea and health-related behaviors is well-documented. In our study, eating breakfast and exercising regularly were associated with lower dysmenorrhea scores. Skipping breakfast was consistently associated with higher levels of dysmenorrhea in our study and consistent with other studies addressing nutrition and dysmenorrhea [50, 51]. Physical activity and regular exercise throughout the month have been shown to reduce dysmenorrhea by enhancing pelvic blood circulation and can improve psychological well-being [52].

The literature shows a strong link between dysmenorrhea and menstrual characteristics, including AAM, bleeding duration, frequencey, and intensity [5, 7, 8, 30, 36, 39]. Most research has connected dysmenorrhea to an earlier AAM, whereas some have linked it to the length of time since menarche (i.e., gynecological age) [53]. Dysmenorrhea is also strongly associated with the heavy menstrual flow [5]. These findings are consistent with our results which showed that menstrual irregularity and menstrual pain are frequently connected and that menstrual irregularity tends to enhance menstrual pain. This implies that effective management of dysmenorrhea requires a holistic approach rather than only focusing on the pain when assessing girls’ menstrual health.

Adopting a holistic approach is essential and implies integrating health education, support, infrastructure, and measures to combat the social taboos and stigma surrounding menstruation and facilitate health seeking when needed [54]. This is important, especially since women and girls usually do not seek health care for dysmenorrhea, assuming it is normal or hindered by the socio-cultural barriers related to menstruation [5, 7]. Furthermore, this approach for menstrual health must be incorporated into the girls’ life course that tackles health risk exposures and link them to future health [55]. This is important when considering that girls live with menstruation throughout their reproductive lives. Risk factors and health issues emerging during adolescence or earlier can affect future reproductive health and general well-being [55]. This is quite evident in the case of secondary dysmenorrhea which includes endometriosis, [56, 57]known to affect between 5 and 15% of women and girls [56]. Endometriosis causes chronic pain and fertility problems. [10, 56, 58]Thus, assessing dysmenorrhea and menstrual irregularities early enough can help support women’s and girls’ health and prevent future complications [58].

### Strengths and limitations

A key strength of this study is the breadth of the studied topic that has not been explored adequately in the two study areas, in addition to its large sample size and representativeness of all Palestine refugee camps in the West Bank and Jordan. The sample was proportionally distributed throughout all camps. After data collection, the proportion of adolescent girls in each refugee camp was accounted for by comparing it with official statistics. A sensitivity analysis comparing weighted and unweighted results showed no significant difference. Consequently, we used unweighted data to prevent bias from weighting. In addition, we applied a multi-dimensional measure to assess dysmenorrhea, representing more realistic levels and accurate estimates than a single query. To ensure reliability, the field workers were trained and piloted the questionnaire before data collection started. However, this study did not cover some broader risk factors for dysmenorrhea, including girls’ education, family history, type of medication used, and lifestyle habits that are worth exploring in future studies.

## Conclusion

The results of our study are consistent with the expanding body of literature addressing menstrual health and describing normal menstrual characteristics and irregularities, such as dysmenorrhea and correlated factors. However, in previous literature, little attention has been paid to how these patterns or irregularities should be addressed at the policy or research levels in relation to pain diagnosis/evaluation and management. The available research is sporadic, fragmented, and predominantly descriptive, giving insufficient data for policy formulation. More effort should be invested in addressing menstrual patterns and irregularities systematically and efficiently.

It is evident that adolescent girls and their families require improved menstrual awareness campaigns and education programs, particularly in relation to everyday menstrual activities. Our findings suggest that regular physical activity and eating breakfast habitually might ease dysmenorrhea; hence, disseminating such information would be helpful. Additionally, previous research has reported that caffeine consumption, consuming spicy or high-fat food, poor sleeping quality, obesity, and exposure to high levels of stress and anxiety might increase dysmenorrhea levels [8, 45, 50, 59], which should also be conveyed to adolescent girls and their families. Such health education messages need to be reinforced on multiple socio-ecological levels, notably in schools and health institutions, where all can be targeted, regardless of whether they seek health care through primary or secondary prevention [57, 60, 61].

Furthermore, for better menstrual health at the population level, clear, agreed-upon practical definitions for menstrual characteristics and disturbances are needed. These should target menstruation from different dimensions, including health, education, human rights, and gender equality. A clear, unified operational definition for dysmenorrhea that is multi-dimensional and age-specific is needed. This will aid in identifying and tracking dysmenorrhea trends, as well as identifying risk factors for distinct groups.

## Electronic supplementary material

Below is the link to the electronic supplementary material.

Supplementary Material 1

Supplementary Material 2

## Data Availability

Data is available upon appropriate request, however we reserve exclusive use of the study’s primary outcomes until their publication. You can receive all accessible data by contacting the corresponding author at rghandour@birzeit.edu.

## Footnotes

1 *Age, age at menarche and number of bleeding days were analyzed as continuous variables and correlation coefficient was reported. Write what variables have been adjusted for*.

2 *Correlation coefficient*.

## Abbreviations

AAM: Age at Menarche
MDS: Mean Dysmenorrhea Score
MENA: Middle East and North Africa
oPt: occupied Palestinian territory
UNRWA: United Nations Relief and Works Agency in the Near East
WaLIDD: Working ability, Location, Intensity, Duration of pain Dysmenorrhea
WASH: Water, Sanitation, and Hygiene
