# Supplementary material for: Menstrual characteristics and dysmenorrhea among Palestinian adolescent refugee camp dwellers in the West Bank and Jordan: a cross-sectional study
Source: Arch Public Health. 2023 Mar 30;81:47. doi: 10.1186/s13690-023-01059-6 (PMC10061948; doi:10.1186/s13690-023-01059-6)
Supplement: Supplementary file 1 — Supplementary Material 1 [file 13690_2023_1059_MOESM1_ESM.docx]

**Supplement 1: Sample size calculation and sample flowchart**

**Sample size calculation**

The sample was a stratified random sample with proportional allocation. The sample size was calculated based on a standard simple random design and then corrected for design effect (clustering) using the following formula:

$$n=\frac{{1.96}^{2}*r\left( 1-r \right)*deff}{({0.15r)}^{2}}$$

Where

**r**: is the prevalence of anemia, the lowest expected indicator in the bigger project from which the data was obtained (See methods section)

**deff**: design effect to account for the cluster sampling (assigned as 2)

**0.15**: margin of error tolerated at 95% confidence interval

The sample was calculated for each country independently given the differnce in anemia level:

| ***West Bank of Palestine:***  $n=\frac{{1.96}^{2}*0.19\left( 1-0.19 \right)*2}{({0.15*0.19)}^{2}}$ = 1456 | ***Jordan:***  $n=\frac{{1.96}^{2}*0.26\left( 1-0.26 \right)*2}{({0.15*0.26)}^{2}}$*=* 972 |
| --- | --- |

The total sample size calculated was 2428. However, we obtained data from 2949 girls, and 2737 girls met the inclusion requirements for this paper's study topic and were ready for the final analysis (see flowchart below for details on how participants were approached and included).

There were two levels of recruitment: initially, the household, and then adolescent girls. We first requested the participation of female household heads, independent of the presence or absence of adolescent females in their households. Then, after filling out the roster information, we obtain permission to interview all adolescent girls residing in the selected household if the family has any adolescent girls in this age group living in the household. Then, we sought the girls' own approval.
