# Supplementary material for: Menstrual characteristics and dysmenorrhea among Palestinian adolescent refugee camp dwellers in the West Bank and Jordan: a cross-sectional study
Source: Arch Public Health. 2023 Mar 30;81:47. doi: 10.1186/s13690-023-01059-6 (PMC10061948; doi:10.1186/s13690-023-01059-6)
Supplement: Supplementary file 2 — Supplementary Material 2 [file 13690_2023_1059_MOESM2_ESM.docx]

**Letter to Editor – Aims and Scope statement**

- **What is known up to now?**

Menstrual health is integral to adolescent girls' reproductive health in their transition to adulthood. Globally, adolescent girls may suffer from a wide range of menstrual irregularities and a very high rate of dysmenorrhea (menstrual pain). However, available research is fragmented and disintegrated with limited agreed-upon definitions for measuring such health conditions. Given the growing global research on menstrual health in recent decades, still, such research is very limited in the Eastern Mediterranean region, including Arab countries. We know almost nothing about Palestinian adolescents' menstrual health and irregularities, especially those who reside in refugee camps and have poor health outcomes due to their political instability and socio-economic circumstances.

- **What does the study add?**

Adolescent girls living in Palestinian refugee camps in the West Bank of the occupied Palestinian territory and Jordan reported high levels of regular menstrual cycles in terms of duration, length, and intensity of bleeding. They did, however, suffer from extremely high levels of dysmenorrhea (menstrual pain), which was strongly associated with menstrual irregularities. The severity of their dysmenorrhea was likewise associated with the girls' low socio-economic status and poor health habits. Higher levels of dysmenorrhea was linked to skipping breakfast regularly and low levels of regular physical activity patterns. Dysmenorrhea was a debilitating event for these adolescent girls as reflected by the multi-dimensional scale used to measure it (Working ability, Location, Intesity, Days of pain Dysmenorrhea (WaLIDD) scale). Such findings necessitate a holistic approach to menstrual health and dysmenorrhea that goes beyond pain management.

**Implication of the study for public health practice or policy and or for future research**

According to this study's findings, dysmenorrhea needs to be better addressed as part of a holistic approach to menstrual health, reproductive health, and general well-being. This is important, especially for adolescents transitioning to adulthood who suffer from high levels of stressors that might impact their physical and mental health.

Menstruation, including menstrual disorders, needs to be addressed more effectively by households, schools, and health care professionals. At these different socio-ecological levels, collaborative efforts are required to provide health education regarding menstrual health and the assessment and management of menstrual abnormalities, including dysmenorrhea. This should begin as early as possible during adolescence and needs a strong political will and commitment from policymakers in the field.

At the research level, it is essential to construct socially, culturally, and age-appropriate, consistent definitions and measurement tools for menstrual irregularities and disorders, including dysmenorrhea, to understand their prevalence and associated factors and allow for comparison between different population groups.
